# Development of a genetic evaluation for hair shedding in American Angus cattle to improve thermotolerance

**DOI:** 10.1186/s12711-020-00584-0

**Published:** 2020-10-21

**Authors:** Harly J. Durbin, Duc Lu, Helen Yampara-Iquise, Stephen P. Miller, Jared E. Decker

**Affiliations:** 1 University of Missouri, Columbia, MO 65211 USA; 2 Angus Genetics Inc., St. Joseph, MO 64506 USA

## Abstract

**Background:**

Heat stress and fescue toxicosis caused by ingesting tall fescue infected with the endophytic fungus *Epichloë coenophiala* represent two of the most prevalent stressors to beef cattle in the United States and cost the beef industry millions of dollars each year. The rate at which a beef cow sheds her winter coat early in the summer is an indicator of adaptation to heat and an economically relevant trait in temperate or subtropical parts of the world. Furthermore, research suggests that early-summer hair shedding may reflect tolerance to fescue toxicosis, since vasoconstriction induced by fescue toxicosis limits the ability of an animal to shed its winter coat. Both heat stress and fescue toxicosis reduce profitability partly via indirect maternal effects on calf weaning weight. Here, we developed parameters for routine genetic evaluation of hair shedding score in American Angus cattle, and identified genomic loci associated with variation in hair shedding score via genome-wide association analysis (GWAA).

**Results:**

Hair shedding score was moderately heritable (*h*^*2*^ = 0.34 to 0.40), with different repeatability estimates between cattle grazing versus not grazing endophyte-infected tall fescue. Our results suggest modestly negative genetic and phenotypic correlations between a dam’s hair shedding score (lower score is earlier shedding) and the weaning weight of her calf, which is one metric of performance. Together, these results indicate that economic gains can be made by using hair shedding score breeding values to select for heat-tolerant cattle. GWAA identified 176 variants significant at FDR < 0.05. Functional enrichment analyses using genes that were located within 50 kb of these variants identified pathways involved in keratin formation, prolactin signalling, host-virus interaction, and other biological processes.

**Conclusions:**

This work contributes to a continuing trend in the development of genetic evaluations for environmental adaptation. Our results will aid beef cattle producers in selecting more sustainable and climate-adapted cattle, as well as enable the development of similar routine genetic evaluations in other breeds.

## Background

At the beginning of the summer, many mammalian species molt thick winter coats in response to changing day length in order to prepare for warmer temperatures [1–6]. There is evidence of quantitative variation in the rate and timing of this yearly shedding across taxa [7, 8], including cattle [9]. In warm climates, cattle that shed their winter coat earlier and more completely have an adaptive advantage over later-shedding herd-mates. Late-shedding cattle will need to partition energy that could have gone towards growth and production towards overcoming heat stress [10]. Economic losses attributable to heat stress cost the U.S. beef cattle industry more than $360 million each year in 2003 [11], which equates to ~ $518 million in 2020 after adjustment for inflation. In the cow-calf sector, a portion of this economic impact is a result of lowered calf weaning weights caused by reduced dam productivity [12]. However, there is currently no national-scale genetic evaluation for heat tolerance. In the United States, much of the beef herd that is at risk of heat stress is also at risk for fescue toxicosis. Tall fescue (*Lolium arundinaceum*) is the most widely available forage in the United States [13], thanks in part to its symbiotic relationship with the endophytic fungus *Epichloë coenophiala*. *E. coenophiala* produces ergot alkaloids that benefit the forage by increasing drought tolerance and pathogen resistance [14], but negatively impact livestock to varying degrees. In cattle, one side-effect of fescue toxicosis is peripheral vasoconstriction, which reduces the animal’s ability to dissipate heat. The ergot alkaloids that cause fescue toxicosis also disrupt the hair follicle growth cycle, which interferes with hair coat shedding and, in turn, further increases the potential for heat stress [15]. Therefore, effective early-summer hair shedding while grazing endophyte-infected (hereafter referred to as “toxic”) tall fescue may also be an indicator of tolerance to fescue toxicosis. One way to mitigate heat stress is through introgression of beneficial alleles from tropically-adapted breeds [16]. However, this can take many generations and may come at the cost of other production traits. An alternative strategy is the exploitation of standing genetic variation in the population of interest. Recently, interest has grown in augmenting national genetic evaluations with predictions of regional adaptability and suitability [17–19], particularly by using novel traits [20]. Here, we develop parameters for a prototype national genetic evaluation of hair shedding in American Angus cattle, a novel trait that directly influences cattle’s ability to dissipate heat. To assess one potential impact of such an evaluation on beef cattle producers, we also demonstrate the relationship between dam hair shedding score and the weaning weight of her calf. This evaluation will aid beef cattle producers in heat-stressed regions in the selection of more sustainable cattle.

## Methods

### Data

**Fig. 1:**
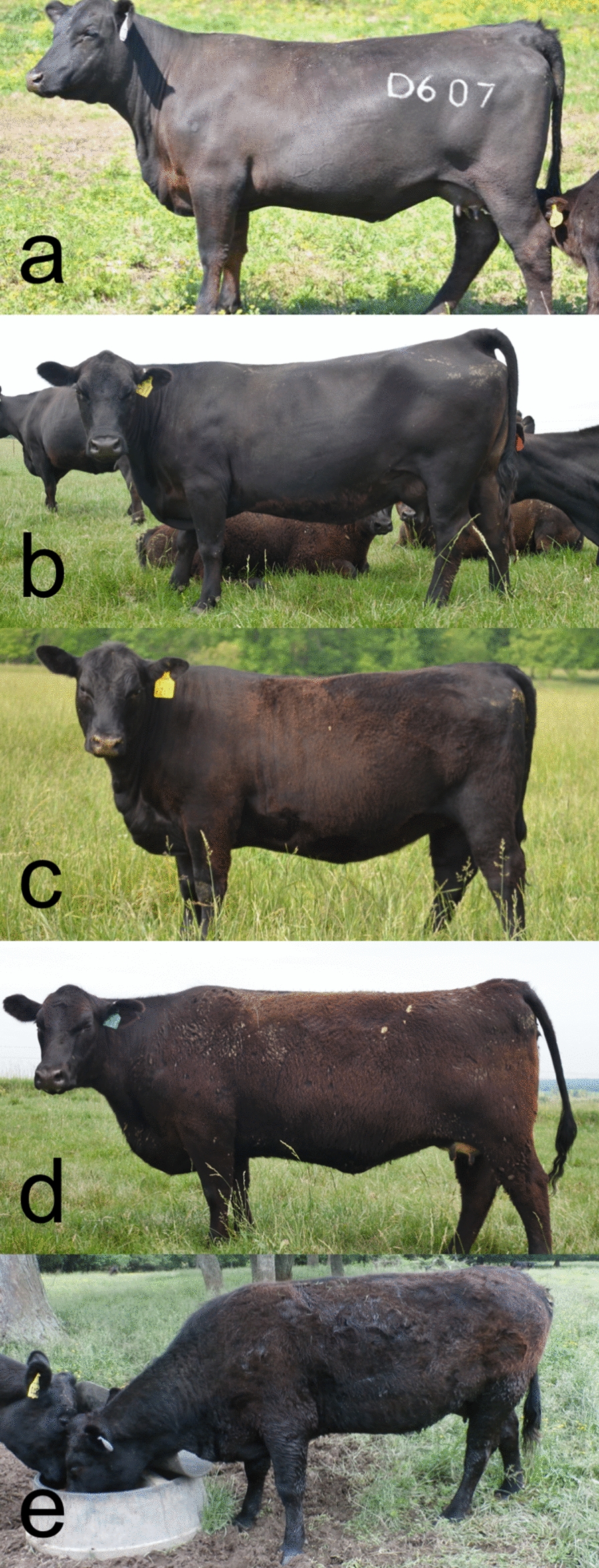
Hair shedding scoring system. Examples of the 1 to 5 visual appraisal hair shedding scoring system used in this research. **a** Score of 1, 0% dead winter coat remaining. **b** Score of 2, approximately 25% of winter coat remaining, typically observed on the lower hindquarter, flank and belly. **c** Score of 3, approximately 50% of winter coat remaining. **d** Score of 4, approximately 75% of winter coat remaining. Hair is typically removed from the head and neck first. **e** Score of 5, 100% winter coat remaining

All data originated from purebred cattle registered in the American Angus Association (AAA) and commercial cattle enrolled in the AAA Breed Improvement Record program. Phenotypic data comprised hair shedding scores recorded by beef cattle producers enrolled in the Mizzou Hair Shedding Project (MU data) between 2016 and 2019 in combination with hair shedding scores collected by technicians in 2011, 2012, 2018, and 2019 as part of Angus Foundation-funded projects at Mississippi State University and North Carolina State University (AGI data). Across all years and both datasets, scores were recorded on 1 day between April 17th and June 30th in the late spring or early summer, with most scores recorded in mid- to late-May. Hair shedding was evaluated using a 1 to 5 visual appraisal scale, where 1 was 0% dead winter coat remaining and 5 was 100% winter coat remaining based on the systems developed by Turner and Schleger [21] and Gray et al. [22] (Fig. 1). While there is variation in the onset of hair shedding between individuals, cattle and other mammals tend to shed from the head towards the tail and from the topline towards the legs [2, 8, 23].

**Fig. 2:**
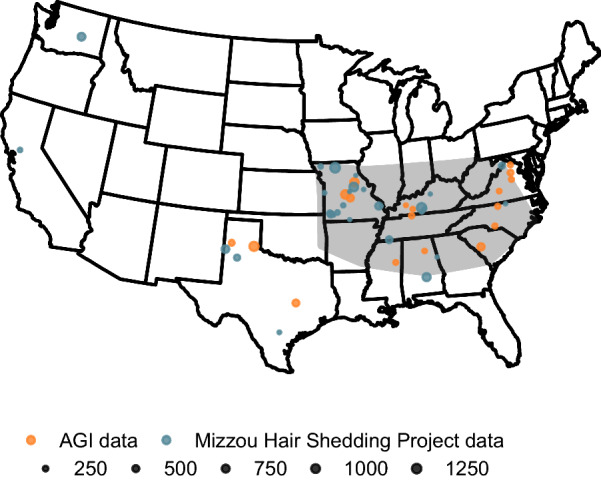
Geographic distribution of animals with hair shedding scores. Hair shedding scores in both the AGI and MU datasets originated primarily from the South and the Fescue Belt. Here, the approximate location of the Fescue Belt is shaded in grey. Size of circles denotes the number of hair shedding scores recorded at that location. Farmers and ranchers in the MU dataset reported whether cattle grazed the predominant endophyte-infected fescue forage or a different forage species

Records were removed when the breeder-reported sex of an animal did not match the sex recorded in the AAA pedigree. Hair shedding scores that originated from male animals comprised less than 1% of the dataset and only female records were retained. Age classifications were assigned to each record based on age in days determined by the AAA-recorded birth date and the date the hair shedding score was recorded. Similar to the system used in the Beef Improvement Federation (BIF) Guidelines for age-of-dam classification [24], age classifications were defined as $\left( {n*365d} \right) - 90d$ to $\left( {\left( {n + 1} \right)*365d} \right) - 90d$, where $n$ is the age classification and $d$ is days. Records where the breeder-reported age in years differed from the calculated age classification by more than 2 years and records from animals younger than 275 days-of-age were removed. When no calving season was reported, it was imputed using the most recent natural birth calving date available in the AAA database prior to the recorded score. When no natural birth calving dates were available, calving season was imputed using the animal’s own birth date. In the AGI data, some animals were scored by multiple scoring technicians on the same day. In these cases, phenotypes recorded on the same animal and the same day were averaged. In the MU data, participating producers were asked to report whether or not (yes or no) animals grazed toxic fescue during the spring of the recording year. Grazing status was not explicitly recorded in the AGI data, but animals scored in Texas were assumed not to have grazed toxic fescue. This resulted in 14,839 scores in the combined, filtered dataset. Among the 8619 individuals included, 49% had between 2 and 6 years of data. Most data came from herds in the Southeast and Fescue Belt (Fig. 2). The mean hair shedding score was slightly higher in the AGI data ($\mu$ = 3.10; $n$ = 6374) compared to the MU data ($\mu$ = 2.65; $n$ = 8465), but the standard deviation was identical in both datasets ($\sigma$ = 1.15).

### Genotypes and imputation

Genotypes for 3898 of the 8619 animals were imputed to a union marker set (${\text{n}}$ = 233,246) of the GGP-F250 genotyping chip and various commercial assays using FImpute v.3.0 [25]. The commercial assays were those used in routine genotyping of Angus cattle for genomic selection purposes, which include ~ 50,000 markers or a lower density panel that can be imputed to ~ 50,000 with sufficient accuracy. Although FImpute provides the capacity to infer the genotypes of un-genotyped animals based on information from relatives, markers were imputed only for genotyped individuals. Prior to imputation, markers with a GenCall score lower than 0.15 were set to missing and individuals with Mendelian error rates higher than 2% had their parents set to missing in the pedigree. The GGP-F250 was designed to genotype functional variants and thus has more variants at low minor allele frequencies [26]. Therefore, no minor allele frequency filter was applied during or after imputation beyond the removal of monomorphic SNPs. Animals and markers with call rates lower than 85% were removed. The resulting marker set consisted of 174,894 autosomal variants.

### Construction of the blended relationship matrix ${\mathbf{H}}^{ - 1}$

In single-step genomic best linear unbiased prediction (BLUP) as used in the AAA National Cattle Evaluation (NCE), relationships between individuals are represented in the matrix ${\mathbf{H}}^{ - 1}$, which is a blended form of the genomic and additive relationship matrices [27], allowing information from both genotyped and non-genotyped animals to be used. ${\mathbf{H}}^{ - 1}$ is calculated as:

$${\mathbf{A}}^{ - 1} + \left[ {\begin{array}{*{20}c} 0 & 0 \\ 0 & {{\mathbf{G}}_{\varvec{w}}^{ - 1} - {\mathbf{A}}_{22}^{ - 1} } \\ \end{array} } \right],$$

where ${\mathbf{A}}^{ - 1}$ represents the inverted pedigree relationship matrix traditionally used to represent relationships, ${\mathbf{A}}_{22}^{ - 1}$ represents the inverted pedigree relationship matrix for the subset of animals with genotypes available, and ${\mathbf{G}}_{\varvec{w}}^{ - 1}$ is the inverted genomic relationship matrix. The genomic relationship matrix was calculated using the VanRaden method [28] and was blended with ${\mathbf{A}}_{22}$ with the default weight of 0.05 using the preGSf90 program [29]. In all subsequent models including a random genetic effect, ${\mathbf{H}}^{ - 1}$ was constructed using the 3-generation pedigree (in total, 17,652 animals; 1987 distinct sires and 9509 distinct dams) in combination with imputed genotypes.

### Effect of age on hair shedding score and contemporary group definition

Understanding how and which environmental factors shape phenotypic variation enables the development of more appropriate contemporary group definitions during genetic evaluation. In order to quantify the effect of animal age on hair shedding score, we fitted age as a categorical fixed effect in a repeated records animal model. Age categories were defined in three ways. First, age in years was fit (i.e. all possible values between 1 and 16). Second, ages were grouped as 1, 2, 3, or other (“four-class model”). Third, age groups were defined according to the guidelines set forth by the BIF for age-of-dam effects on birth weight and weaning weight (i.e., 2, 3, 4, 5-9, 10, 11, 12, 13+; [24]) plus yearlings (“BIF model”). The four-class model and the BIF model were each compared against a null model with no age effect included using Akaike’s Information Criterion (AIC) and likelihood ratio tests. In all three models with age classification fitted as a categorical fixed effect, classifications with fewer than five animals were excluded. These models are summarized below:

$${\mathbf{y}} = {\mathbf{X}}_{1} {\mathbf{b}} + {\mathbf{X}}_{2} {\mathbf{a}} + {\mathbf{Z}}_{1} {\mathbf{u}} + {\mathbf{Z}}_{2} {\mathbf{p}} + {\mathbf{e}},$$

where ${\mathbf{y}}$ is a vector of hair shedding scores; ${\mathbf{b}}$ is a vector of contemporary group effects for each hair shedding score, with contemporary group defined as farm ID, year scored, calving season, score group, and toxic fescue grazing status; ${\mathbf{a}}$ is a vector of age classification effects for each individual (based on age-in-years, BIF classifications, or the four age classes); ${\mathbf{u}}$ is the random additive genetic effect with ${\mathbf{u}} \sim {\text{N}}\left( {{\mathbf{0}},{\mathbf{H}}\sigma_{\text{a}}^{2} } \right)$; ${\mathbf{p}}$ is the random permanent environment effect with ${\mathbf{p}} \sim {\text{N}}\left( {{\mathbf{0}},{\mathbf{I}}\sigma_{\text{pe}}^{2} } \right)$; ${\mathbf{e}}$ is the random residual with ${\mathbf{e}} \sim {\text{N}}\left( {{\mathbf{0}},{\mathbf{I}}\sigma_{\text{e}}^{2} } \right)$; and ${\mathbf{X}}_{1}$, ${\mathbf{X}}_{2}$, ${\mathbf{Z}}_{1}$, and ${\mathbf{Z}}_{2}$ are incidence matrices relating the elements of ${\mathbf{y}}$ to ${\mathbf{b}}$, ${\mathbf{a}}$, ${\mathbf{u}}$, and ${\mathbf{p}}$, respectively.

### Effect of toxic fescue grazing status on hair shedding

Cattle reared in heat-stressed regions but not exposed to endophyte-infected fescue demonstrate similar benefits from early summer hair shedding, but it is unclear if the biological mechanisms that govern hair shedding under fescue toxicosis and heat stress alone are the same. This could have implications for routine genetic evaluation, as it might require that some hair shedding score observations be treated as a separate trait. In order to clarify the relationship between hair shedding score while grazing toxic fescue versus while not grazing toxic fescue, we calculated the covariance and genetic correlation between hair shedding score grazing toxic fescue and not grazing toxic fescue using the bivariate repeated records animal model below:

$${\mathbf{y}}_{{\mathbf{t}}} = {\mathbf{X}}_{{\mathbf{t}}} {\mathbf{b}}_{{\mathbf{t}}} + {\mathbf{Z}}_{{1{\mathbf{t}}}} {\mathbf{u}}_{{\mathbf{t}}} + {\mathbf{Z}}_{{2{\mathbf{t}}}} {\mathbf{p}}_{{\mathbf{t}}} + {\mathbf{e}}_{{\mathbf{t}}} ,$$

where ${\mathbf{y}}$ is a vector hair shedding scores and ${\mathbf{t}}$ is toxic fescue grazing status (yes or no); ${\mathbf{b}}$ is a vector of contemporary group effects for each hair shedding score, with contemporary groups defined as farm ID, year scored, calving season, score group, and age class (yearling, 2-year-old, 3-year-old, or other; based on the results of the age classification analyses above); ${\mathbf{u}}$ is the additive genetic effect and $${\text{Var}}\left( {\mathbf{u}} \right) = \left[ {\begin{array}{*{20}c} {\sigma_{\text{uYes}}^{2} } & {\sigma_{{{\text{uYes}},{\text{uNo}}}} } \\ {\sigma_{{{\text{uNo}},{\text{uYes}}}} } & {\sigma_{\text{uNo}}^{2} } \\ \end{array} } \right] \otimes {\mathbf{H}}$$; ${\mathbf{p}}$ is the permanent environment effect and $${\text{Var}}\left( {\mathbf{p}} \right) = \left[ {\begin{array}{*{20}c} {\sigma_{\text{pYes}}^{2} } & 0 \\ 0 & {\sigma_{\text{pNo}}^{2} } \\ \end{array} } \right] \otimes {\mathbf{I}}$$; ${\mathbf{e}}$ is the random residual and $${\text{Var}}\left( {\mathbf{e}} \right) = \left[ {\begin{array}{*{20}c} {\sigma_{\text{eYes}}^{2} } & {\sigma_{{{\text{eYes}},{\text{eNo}}}} } \\ {\sigma_{{{\text{eNo}},{\text{eYes}}}} } & {\sigma_{\text{eNo}}^{2} } \\ \end{array} } \right] \otimes {\mathbf{I}}$$; and ${\mathbf{X}}$, ${\mathbf{Z}}_{1}$, and ${\mathbf{Z}}_{2}$ are incidence matrices relating the elements of ${\mathbf{y}}$ to ${\mathbf{b}}$, ${\mathbf{u}}$, and ${\mathbf{p}}$, respectively.

In addition, we fitted a univariate model with toxic fescue grazing status included as a categorical fixed effect. The goal of this model was to quantify the effect of reported toxic fescue grazing status on hair shedding score:

$${\mathbf{y}} = {\mathbf{X}}_{1} {\mathbf{b}} + {\mathbf{X}}_{2} {\mathbf{f}} + {\mathbf{Z}}_{1} {\mathbf{u}} + {\mathbf{Z}}_{2} {\mathbf{p}} + {\mathbf{e}},$$

where ${\mathbf{y}}$ is a vector of hair shedding scores; ${\mathbf{b}}$ is a vector of contemporary group effects, defined in the same way as the univariate model above; ${\mathbf{f}}$ is the toxic fescue status effect (yes or no); ${\mathbf{u}}$ is the additive genetic effect with ${\mathbf{u}} \sim {\text{N}}\left( {{\mathbf{0}},{\mathbf{H}}\sigma_{\text{a}}^{2} } \right)$; ${\mathbf{p}}$ is the permanent environment effect with ${\mathbf{p}} \sim {\text{N}}\left( {{\mathbf{0}},{\mathbf{I}}\sigma_{\text{pe}}^{2} } \right)$; ${\mathbf{e}}$ is the random residual with ${\mathbf{e}} \sim {\text{N}}\left( {{\mathbf{0}},{\mathbf{I}}\sigma_{\text{e}}^{2} } \right)$; and ${\mathbf{X}}_{1}$, ${\mathbf{X}}_{2}$, ${\mathbf{Z}}_{1}$, and ${\mathbf{Z}}_{2}$ are incidence matrices relating the elements of ${\mathbf{y}}$ to ${\mathbf{b}}$, ${\mathbf{f}}$, ${\mathbf{u}}$, and ${\mathbf{p}}$, respectively.

In both models, only females with known toxic fescue grazing status were retained for analysis. Contemporary groups with fewer than five animals or no variation were discarded, resulting in 5832 observations from cattle grazing toxic fescue and 4197 observations from cattle not grazing toxic fescue. Three hundred ninety-six animals had observations over multiple years both grazing and not grazing toxic fescue.

### Genetic parameters, breeding values, and estimated bias

Variance components, heritability, repeatability, and breeding values were estimated using the univariate repeated records animal model below implemented in AIREMLF90 [29].

$${\mathbf{y}} = {\mathbf{Xb}} + {\mathbf{Z}}_{1} {\mathbf{u}} + {\mathbf{Z}}_{2} {\mathbf{p}} + {\mathbf{e}},$$

where ${\mathbf{y}}$ is a vector of hair shedding scores; ${\mathbf{b}}$ is the contemporary group effect; ${\mathbf{u}}$ is the additive genetic effect with ${\mathbf{u}} \sim {\text{N}}\left( {{\mathbf{0}},{\mathbf{H}}\sigma_{\text{a}}^{2} } \right)$; ${\mathbf{p}}$ is the permanent environment effect with ${\mathbf{p}} \sim {\text{N}}\left( {{\mathbf{0}},{\mathbf{I}}\sigma_{\text{pe}}^{2} } \right)$; ${\mathbf{e}}$ is the random residual with ${\mathbf{e}} \sim {\text{N}}\left( {{\mathbf{0}},{\mathbf{I}}\sigma_{\text{e}}^{2} } \right)$; and ${\mathbf{X}}$, ${\mathbf{Z}}_{1}$, and ${\mathbf{Z}}_{2}$ are incidence matrices relating the elements of ${\mathbf{y}}$ to ${\mathbf{b}}$, ${\mathbf{u}}$, and ${\mathbf{p}}$, respectively.

The definition of contemporary groups used in this final prediction model was informed by the results of the age classification and toxic fescue grazing status analyses above. It included a combination of farm, year scored, calving season (spring or fall), toxic fescue grazing status (yes or no), age group (yearling, 2-year-old, 3-year-old, or other), and score group. In herds where cattle were scored for hair shedding over more than 1 day, the score group was determined using a 7-day sliding window to maximize the number of animals per contemporary group. In the future, it will be recommended that producers score all cattle for hair shedding within a week of one another to maximize the size of contemporary groups. Although yearling heifers have not yet experienced the stress of pregnancy, calving season/birth season is a good proxy for management group in the absence of breeder-reported codes. Therefore, “calving season” was included in the contemporary group definition for all animals regardless of reproductive status. Contemporary groups with fewer than five animals or no variation were dropped. This resulted in 14,438 total scores from 8449 animals in 395 contemporary groups.

In order to evaluate model bias, we estimated breeding values in ten separate iterations, excluding all phenotypes for a randomly selected 25% of animals. These “partial” breeding values were then compared to breeding values obtained via the “whole” model including all possible information using the “LR method” parameters proposed by Legarra and Reverter [30]. First, we calculated the absolute difference between whole breeding values and partial breeding values for the validation set, or animals whose phenotypes were excluded ($d_{w,p}^{v}$) and the reference set, or animals whose phenotypes were not excluded ($d_{w,p}^{r}$). The expectation of this value is zero in the absence of bias, where bias is introduced by incorrect estimation of the genetic trend. Next, we regressed whole breeding values on partial breeding values for both validation ($b_{w,p}^{v}$) and reference ($b_{w,p}^{r}$) sets. In this model, deviations of the slope from 1 are suggestive of dispersion. Finally, we calculated the correlation between partial and whole breeding values ($\rho_{p,w} = \frac{{cov\left( {\hat{\mu }_{p} ,\hat{\mu }_{w} } \right)}}{{ \sqrt{var(\hat{\mu }_{p}) var (\hat{\mu }_{w})} }}$) within the validation and reference sets, where the correlation within the validation set ($\rho_{p,w}^{v}$) is a metric of prediction accuracy.

### Weaning weight

The effects of heat stress on pre-weaning growth are well characterized in cattle. Heat stress impacts calf performance most severely via reduced milk production in the dam [12]. Fescue toxicosis induces reduced milk production in a similar fashion [31]. Therefore, we quantified the phenotypic and genetic correlations between hair shedding score and weaning weight.

Weaning weight phenotypes and contemporary group designations came from the weekly growth run of the AAA national cattle evaluation (NCE). Prior to entering the NCE, phenotypes were adjusted for age-of-dam effects as used in the AAA weekly NCE and to 205 days-of-age. Weaning weight data were retrieved for: (1) own weaning weight of cows with at least one hair shedding score recorded, (2) all of cow’s recorded calves, (3) cow’s weaning weight contemporary group peers, and (4) all of their recorded calves’ weaning weight contemporary group peers. Weaning weights from animals born via embryo transfer and contemporary groups with fewer than five animals or no variation were excluded, resulting in 40,794 total weaning weights and 14,039 total hair shedding score records. Of the 45,420 phenotyped animals retained for analysis, 3850 had both a recorded weaning weight and at least one hair shedding score. Furthermore, 6448 dams had both hair shedding scores and calf weights recorded in at least 1 year (n = 9092 score/weight pairs).

Conceivably, environmental factors that affect a dam’s hair shedding performance could also affect the direct weaning weight of her calf and her maternal effect on the calf’s growth, creating a residual covariance between the two traits. In order to reflect this covariance, a bivariate model was fitted in which a direct hair shedding score effect was modeled for the cow, a direct weaning weight effect was modeled for the calf, and a maternal weaning weight effect was modeled for the cow. In practice, this model was implemented by fitting a maternal genetic effect for hair shedding, no direct genetic effect of hair shedding (no genetic effect of the calf on the hair shedding score of its dam), and direct and maternal genetic effects for weaning weight. This model created a direct tie between a dam’s hair shedding score and the calf she weaned that year, which reflects more accurately the relationship of interest and is similar to models used to assess the correlations between weaning weight and actual milk yield [32]. For cows with a hair shedding score but no calf weaning weight reported during the scoring year, a “dummy calf” with a weaning weight set to missing and unknown sire was created. This model was fitted three separate times: once including only dams explicitly recorded to have been grazing toxic fescue, once including only dams explicitly recorded to have not been grazing toxic fescue, and once with all available data.

$$\left[ {\begin{array}{*{20}c} {{\mathbf{y}}_{{{\mathbf{HS}}}} } \\ {{\mathbf{y}}_{{{\mathbf{WW}}}} } \\ \end{array} } \right] = \left[ {\begin{array}{*{20}c} {{\mathbf{X}}_{{{\mathbf{HS}}}} } & 0 \\ 0 & {{\mathbf{X}}_{{{\mathbf{WW}}}} } \\ \end{array} } \right]\left[ {\begin{array}{*{20}c} {{\mathbf{b}}_{{{\mathbf{HS}}}} } \\ {{\mathbf{b}}_{{{\mathbf{WW}}}} } \\ \end{array} } \right] + \left[ {\begin{array}{*{20}c} 0 & 0 \\ 0 & {{\mathbf{Z}}_{{1_{{{\mathbf{WW}}}} }} } \\ \end{array} } \right]\left[ {\begin{array}{*{20}c} 0 \\ {{\mathbf{u}}_{{{\mathbf{WW}}}} } \\ \end{array} } \right]$$

$$+ \left[ {\begin{array}{*{20}c} {{\mathbf{Z}}_{{2_{{{\mathbf{HS}}}} }} } & 0 \\ 0 & {{\mathbf{Z}}_{{2_{{{\mathbf{WW}}}} }} } \\ \end{array} } \right]\left[ {\begin{array}{*{20}c} {{\mathbf{m}}_{{{\mathbf{HS}}}} } \\ {{\mathbf{m}}_{{{\mathbf{WW}}}} } \\ \end{array} } \right] + \left[ {\begin{array}{*{20}c} {{\mathbf{Z}}_{{3_{{{\mathbf{HS}}}} }} } & 0 \\ 0 & {{\mathbf{Z}}_{{3_{{{\mathbf{WW}}}} }} } \\ \end{array} } \right]\left[ {\begin{array}{*{20}c} {{\mathbf{mpe}}_{{{\mathbf{HS}}}} } \\ {{\mathbf{mpe}}_{{{\mathbf{WW}}}} } \\ \end{array} } \right] + \left[ {\begin{array}{*{20}c} {{\mathbf{e}}_{{{\mathbf{HS}}}} } \\ {{\mathbf{e}}_{{{\mathbf{WW}}}} } \\ \end{array} } \right],$$

where ${\mathbf{y}}_{{\mathbf{t}}}$ is the phenotype and ${\mathbf{t}}$ is the trait (hair shedding score (HS) or weaning weight (WW)); ${\mathbf{b}}_{{\mathbf{t}}}$ is the contemporary group effect; ${\mathbf{u}}_{{\mathbf{t}}}$ is the calf genetic effect (fit only for weaning weight) and $${\text{Var}}\left( {\mathbf{u}} \right) = \left[ {\begin{array}{*{20}c} 0 & 0 \\ 0 & {\sigma_{\text{uWW}}^{2} } \\ \end{array} } \right] \otimes {\mathbf{H}}$$, where $\sigma_{\text{uWW}}^{2}$ represents the genetic variance for the calf direct effect of weaning weight; ${\mathbf{m}}_{{\mathbf{t}}}$ is the cow genetic effect and $${\text{Var}}\left( {\mathbf{m}} \right) = \left[ {\begin{array}{*{20}c} {\sigma_{\text{mHS}}^{2} } & {\sigma_{{{\text{mHS}},{\text{mWW}}}} } \\ {\sigma_{{{\text{mWW}},{\text{mHS}}}} } & {\sigma_{\text{mWW}}^{2} } \\ \end{array} } \right] \otimes {\mathbf{H}}$$, where $\sigma_{\text{mHS}}^{2}$ represents the genetic variance for hair shedding and $\sigma_{\text{mWW}}^{2}$ represents the genetic variance for the cow maternal effect of weaning weight; ${\mathbf{mpe}}_{{\mathbf{t}}}$ is the cow permanent environment effect and $${\text{Var}}\left( {{\mathbf{mpe}}} \right) = \left[ {\begin{array}{*{20}c} {\sigma_{\text{mpeHS}}^{2} } & {\sigma_{{{\text{mpeHS}},{\text{mpeWW}}}} } \\ {\sigma_{{{\text{mpeWW}},{\text{mpeHS}}}} } & {\sigma_{\text{mpeWW}}^{2} } \\ \end{array} } \right] \otimes {\mathbf{I}}$$, where $\sigma_{\text{mpeHS}}^{2}$ represents the permanent environmental variance for hair shedding and $\sigma_{\text{mpeWW}}^{2}$ represents the permanent environmental variance for the maternal effect of weaning weight; ${\mathbf{e}}_{{\mathbf{t}}}$ is the random residual and $${\text{Var}}\left( {\mathbf{e}} \right) = \left[ {\begin{array}{*{20}c} {\sigma_{\text{eHS}}^{2} } & {\sigma_{{{\text{eHS}},{\text{eWW}}}} } \\ {\sigma_{{{\text{eWW}},{\text{eHS}}}} } & {\sigma_{\text{eWW}}^{2} } \\ \end{array} } \right] \otimes {\mathbf{I}}$$; and ${\mathbf{X}}$, ${\mathbf{Z}}_{1}$, ${\mathbf{Z}}_{2}$, and ${\mathbf{Z}}_{3}$ are incidence matrices relating the elements of ${\mathbf{y}}$ to ${\mathbf{b}}$, ${\mathbf{u}}$, ${\mathbf{m}}$, and ${\mathbf{mpe}}$, respectively.

We also evaluated the phenotypic relationship between dam hair shedding score and calf weaning weight using the subset of 6448 dams with both hair shedding scores and calf weights recorded in at least 1 year. We did this by calculating the estimated change in calf weaning weight as a function of dam hair shedding score using four separate simple linear regression models. In the first two models, unadjusted calf weaning weight was regressed on unadjusted dam hair shedding score. Using weaning weight unadjusted for age in days captures increased gain from an earlier birth date (older when weighed), which might be an indicator of increased fertility for earlier shedding cows. In the other two models, 205-day, age-of-dam, and contemporary group solution adjusted calf weaning weight was regressed on unadjusted dam hair shedding score. Both the unadjusted weaning weight and adjusted weaning models were fitted separately for all available data, dams explicitly recorded as grazing toxic fescue, and dams explicitly recorded as not grazing toxic fescue.

### Genome-wide association

In order to evaluate the genetic architecture of hair shedding and identify variants that contribute to hair shedding score breeding values, we performed a single-SNP genome-wide association analysis using the SNP1101 v.1 software [33]. The breeding values calculated above using AIREMLF90 were de-regressed and used as the phenotype such that each of the 3783 animals had one record. The de-regressed breeding values were weighted by their reliability $1 - \frac{PEV}{{\sigma_{a}^{2} }}$, where $PEV = \left( {SE^{2} } \right)*\sigma_{e}^{2}$ and $\sigma_{a}^{2}$ and $\sigma_{e}^{2}$ are the estimated additive genetic and residual variances for hair shedding score, respectively. Heritability was constrained to 0.40 and the genomic relatedness matrix used to control for family structure was calculated using the VanRaden method [28].

Using the UMD 3.1 bovine genome assembly [34] coordinates and annotations, we searched genes within 50 kb of SNPs with a genome-wide *q*-value lower than 0.05. The size of our search space was determined based on the density of our marker set, and the resulting gene list was used as input for cluster enrichment analysis within ClueGO v.2.5.6 [35]. KEGG pathways and biological process gene ontologies with at least four associated genes were considered for search terms. We also searched for protein–protein interaction between genes in our list using STRING v.10 [36], considering co-expression, experimental data, and curated databases as active interaction sources.

## Results

### Effect of age on hair shedding score and contemporary group definition

**Fig. 3:**
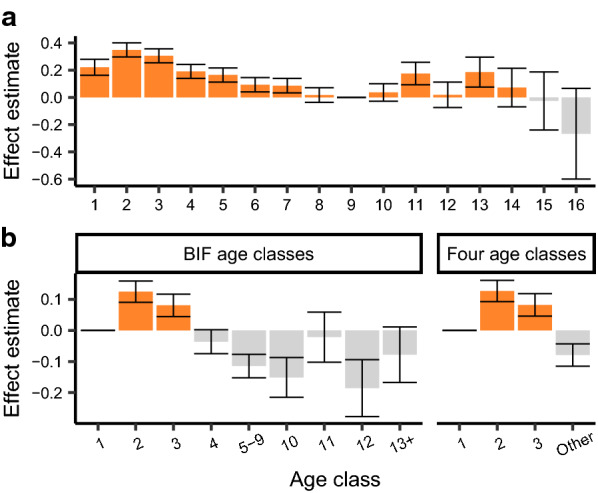
Estimates of the effect of age on hair shedding score. **a** The effect of age in years on hair shedding score appears to be non-linear and follows a U-shaped pattern. **b** Comparison of effect estimates using BIF age-of-dam classifications or four age classes. Error bars represent standard error. Age groups with at least five observations are plotted

The results of the age-in-years model suggest a non-linear effect of age with larger effect sizes in 2-year-old, 3-year-old cows, yearlings, and old cows relative to mature cows (Fig. 3a). Both the BIF age class model and the four age class model had lower AIC values than the null model with no age effect (38912.38, 38906.17, and 38983.31 respectively). Likelihood ratio test results indicate a better fit of the four class model over the null ($- { \log }10\left( p \right)$ = 8.899) and no improvement in model fit using BIF age classes over four age classes ($- { \log }10\left( p \right)$= 0). The power of contemporary grouping is undermined by over-parameterization, which can result in fewer animals per contemporary group. Therefore, we chose to classify age using the simpler four age class definition in all downstream analyses where contemporary group was fitted as a fixed effect in order to maximize contemporary group size.

### Effect of toxic fescue grazing status on hair shedding

When treated as separate traits, hair shedding while grazing and not grazing toxic fescue had similar heritability estimates (Table 1) and a high genetic correlation (*r*_*g*_= 0.93). Furthermore, the Pearson correlation between breeding values grazing and not grazing toxic fescue was 0.99. The total phenotypic variation in hair shedding grazing toxic fescue was slightly higher than hair shedding not grazing toxic fescue, which suggests that reduced peripheral blood flow caused by fescue toxicosis is more detrimental to hair shedding than heat stress alone (Table 1). The fixed-effect model solutions support this conclusion ($\varvec{\beta}_{\varvec{f}}$ = 0 vs. − 0.59 hair shedding score units for grazing and not grazing toxic fescue, respectively). Furthermore, the estimated permanent environment effect (and therefore estimated repeatability, *r*) was much higher for hair shedding while grazing toxic fescue (Table 1).

**Table 1:** Comparison of genetic parameters estimated using cattle grazing and not grazing toxic fescue

	Bivariate model				Univariate model
	$\varvec{\sigma}_{\varvec{P}}^{2}$	$\varvec{\sigma}_{\varvec{A}}^{2}$	***h***^***2***^	***r***	***β***_***f***_
Grazing toxic fescue	0.90	0.38	0.40	0.45	0
Not grazing toxic fescue	0.95	0.30	0.34	0.34	− 0.59 hair shedding score units

The estimated phenotypic variance ($\sigma_{P}^{2}$), and repeatability (*r*) from a bivariate model and fixed effect of grazing versus not grazing fescue from a univariate model. Additive genetic variance, heritability, and repeatability are higher for hair shedding recorded while grazing toxic fescue when treated as a different trait from hair shedding while not grazing toxic fescue. When fescue grazing status is fit as a fixed effect in a univariate model, the estimated effect of toxic fescue on hair shedding score (*β*_*f*_) is also higher (i.e. later shedding animals)

### Genetic parameters, breeding values, and estimated bias

Using all available data, the estimated narrow-sense heritability ($\frac{{\sigma_{a}^{2} }}{{\sigma_{a}^{2} + \sigma_{pe}^{2} + \sigma_{e}^{2} }}$) was 0.40 with an approximate standard error of 0.018. Likewise, the estimated repeatability ($\frac{{\sigma_{a}^{2} + \sigma_{pe}^{2} }}{{\sigma_{a}^{2} + \sigma_{pe}^{2} + \sigma_{e}^{2} }}$) was 0.44 with an approximate standard error of 0.012. These estimates are similar to those previously reported in Angus cattle based on pedigree relatedness [22].

**Fig. 4:**
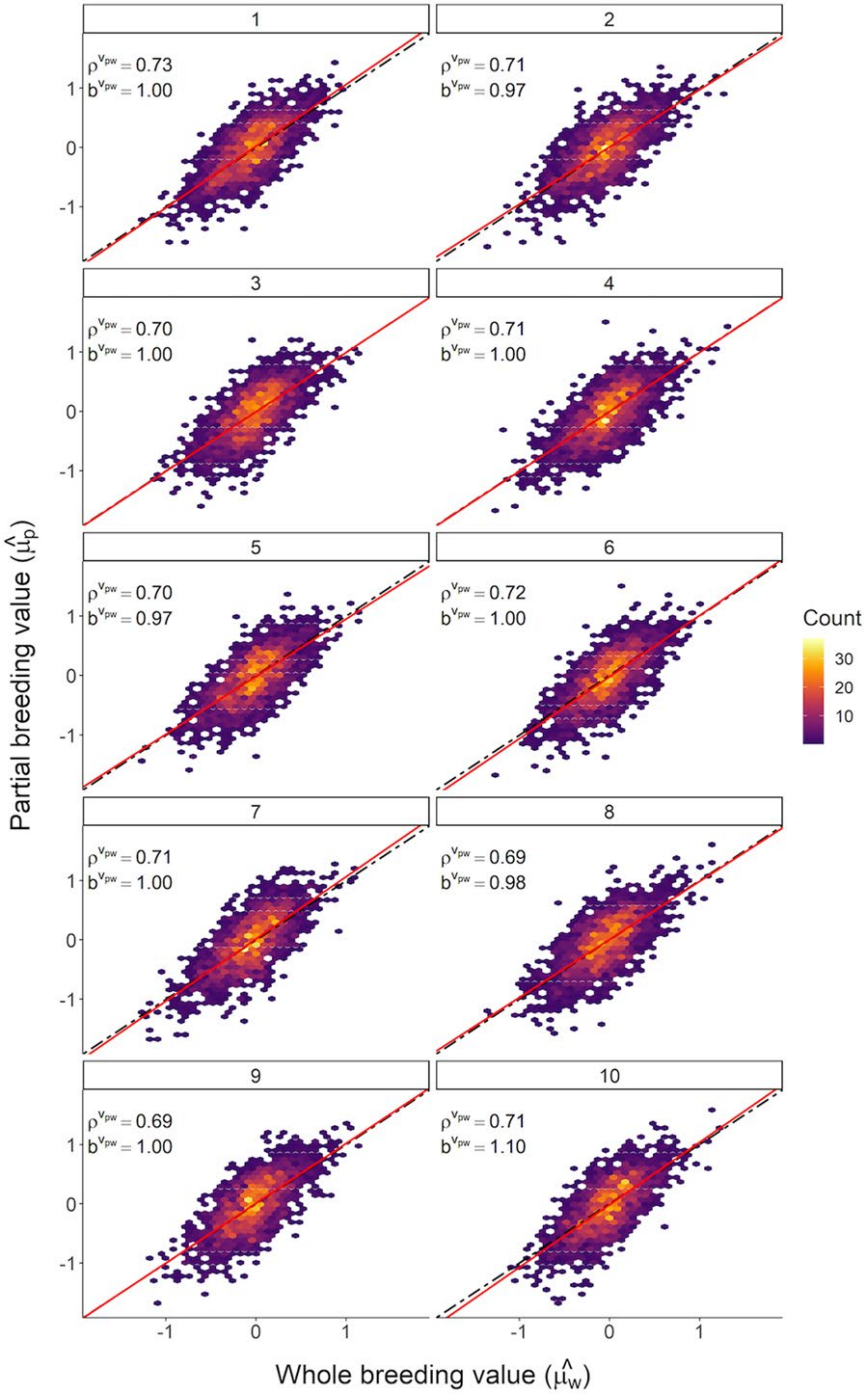
Linear regression evaluation of breeding values. Comparison of breeding values estimated using all available data ($\hat{\mu }_{w}$) and breeding values estimated using a reduced dataset ($\hat{\mu }_{p}$) across ten iterations within validation animals. The solid red line represents $b_{pw}^{v}$ and the dotted black line represents the expectation of $b_{pw}^{v}$ = 1 in the absence of dispersion

Across ten iterations, $d_{p,w}^{v}$ averaged 0.25, ranging from 0 to 1.48. In the absence of bias introduced by incorrect estimation of the genetic trend, this value is expected to be zero. Estimates of $b_{p,w}^{v}$ ranged from 0.96 to 1.05, which suggests minimal dispersion of breeding values (Fig. 4). Prediction accuracy ($\rho_{p,w}^{v}$) ranged from 0.70 to 0.73.

### Relationship between hair shedding and weaning weight

All three bivariate models suggest a moderately negative genetic correlation between weaning weight and hair shedding score. In the model using all available data, the estimated *r*_*g*_ between the maternal component of weaning weight and hair shedding was − 0.19 (Table 2). When the data were stratified by dam toxic fescue grazing status, this estimate increased slightly in magnitude for both grazing and not grazing toxic fescue (*r*_*g*_ = − 0.25 and − 0.28, respectively). For dams not grazing toxic fescue, the *r*_*g*_ between the direct and maternal effect of weaning weight fell near commonly reported estimates (*r*_*g*_ = − 0.29; [37]) but was much higher for dams grazing toxic fescue (*r*_*g*_ = − 0.63) and for all possible dams (*r*_*g*_ = − 0.43) (Table 2). The *r*_*g*_ between the direct effect of weaning weight and hair shedding ranged from − 0.10 (dams not grazing toxic fescue) to − 0.03 (all possible data) to 0 (dams grazing toxic fescue).

**Table 2:** Estimated genetic correlations between dam hair shedding and calf weaning weight

	Weaning weight (direct)	Weaning weight (maternal)
All available data		
Hair shedding	− 0.03 (0.055)	− 0.19 (0.066)
Weaning weight (direct)		− 0.43 (0.050)
Grazing toxic fescue		
Hair shedding	0.01 (0.080)	− 0.25 (0.104)
Weaning weight (direct)		− 0.63 (0.071)
Not grazing toxic fescue		
Hair shedding	0.10 (0.091)	− 0.28 (0.097)
Weaning weight (direct)		− 0.29 (0.097)

Genetic correlation estimates between hair shedding, the direct effect of weaning weight, and the maternal effect of weaning weight vary across toxic fescue grazing statuses with approximated standard errors in parentheses

**Fig. 5:**
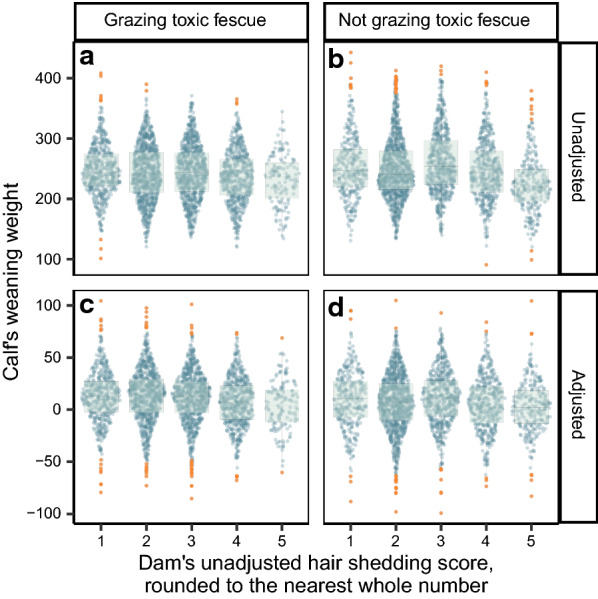
Comparison of dam’s hair shedding score to the weaning weight of her calf. The effect of a dam’s hair shedding score on the unadjusted (**a**, **b**) and adjusted **(c**, **d**) weaning weight of her calf with outlier weaning weights highlighted in orange. Regardless of fescue grazing status, there is very little difference in calf weaning weight between dams with hair shedding scores 1, 2, and 3

In the simple linear models predicting unadjusted weaning weight from dam hair shedding score, unadjusted calf weaning weight was estimated to decrease by 1.30 kg with every unit increase in hair shedding score using all available data, by 3.22 kg for dams grazing toxic fescue and by 5.08 kg for dams not grazing toxic fescue. Slope estimates from the simple linear models predicting adjusted weaning weight from dam hair shedding score were more modest but also negative. Adjusted calf weaning weight was estimated to decrease by 1.45 kg using all possible data, by 2.47 kg among dams grazing toxic fescue, and by 1.11 kg among dams not grazing toxic fescue with every unit increase in hair shedding score (Fig. 5).

### Genome-wide association analysis

**Fig. 6:**
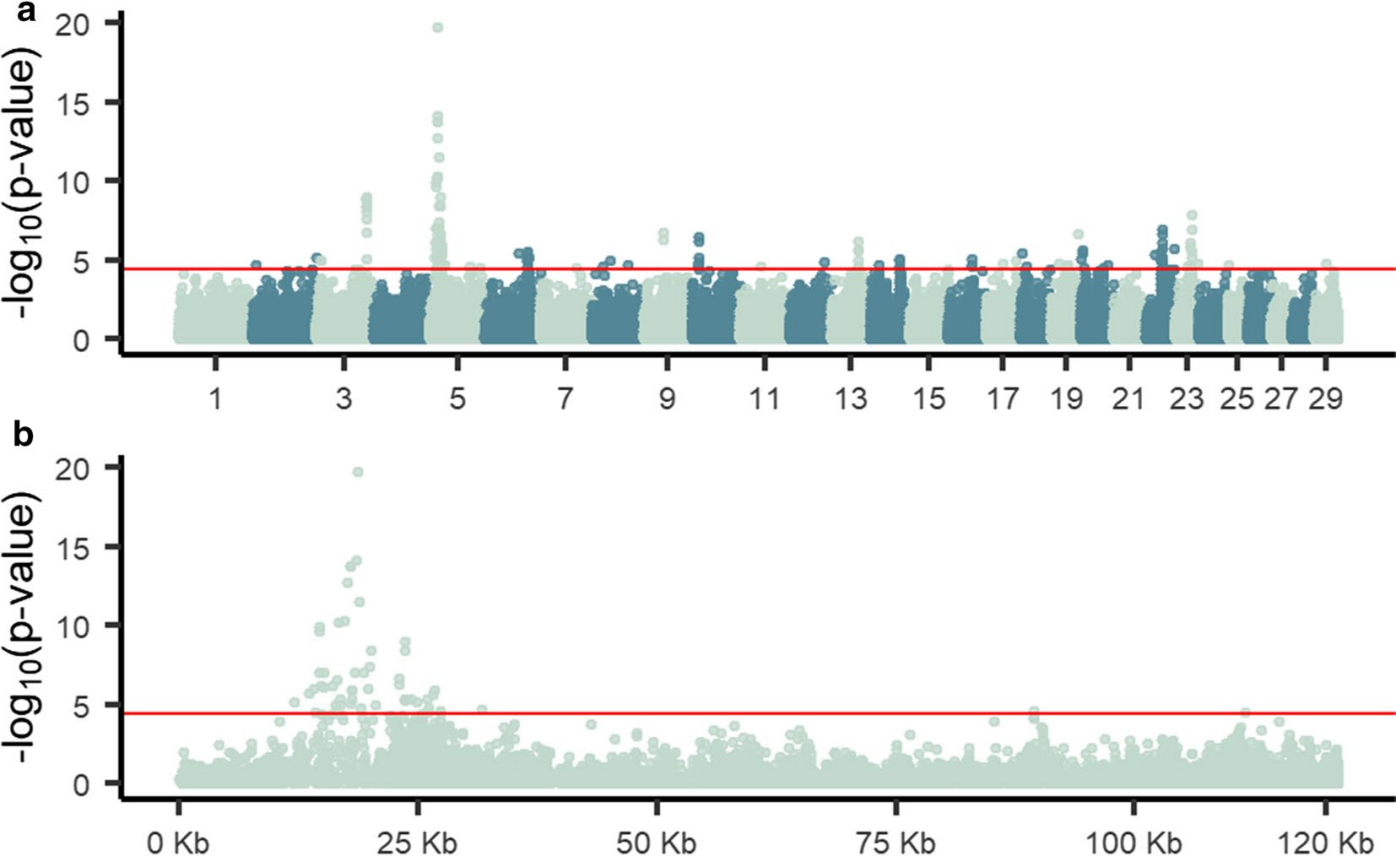
Manhattan plot of variants associated with hair shedding. Using de-regressed hair shedding score breeding values in SNP1101 single-SNP regression, we found 176 variants that are significantly associated with hair shedding (FDR < 0.05, red line) (**a**). Of these 176 variants, 33% reside in a peak on chromosome 5 (**b**)

We found 176 variants that passed the genome-wide false discovery rate threshold of 0.05 and 56 variants that passed the false discovery rate threshold of 0.01 (Fig. 6). Of these 176 variants, 33% are on chromosome 5. Two hundred and six unique genes were found to be within 50 kb of significant variants. The two strongest associations were observed within *CEP290*. Perhaps interestingly, near our largest peak, we identified several members of the KRT gene family (*KRT1*, *KRT3*, *KRT4*, *KRT76*, *KRT77*, *KRT78*, and *KRT79*), which are involved in creating structural epithelial cells like hair.

We found significant enrichment (Benjamini–Hochberg corrected p-value < 0.05) for pathways involved in virus-host interaction, fat cell differentiation, prolactin signalling, cellular response to starvation, vasopressin-regulated water reabsorption, and other biological processes (Table 3). We also found more protein–protein interactions than expected (PPI enrichment p-value = 0.00462) and enrichment for PFAM protein domains “keratin type II head” (FDR = 8.89e−06), “somatotropin hormone family” (FDR = 8.09e−05), and “intermediate filament protein” (FDR = 0.00064).

**Table 3:** Terms significantly associated with genes within 50 kb of hair shedding GWAA variants with FDR < 0.05

Term	Ontology source	p-value	Associated genes
Modulation by virus of host morphology or physiology	GO BP	< 0.001	*ATG7*, *SMAD3*, *VAPB*, *ZC3H12A*
Modification by symbiont of host morphology or physiology	GO BP	< 0.001	*ATG7*, *SMAD3*, *VAPB*, *ZC3H12A*
Modification of morphology or physiology of other organism involved in symbiotic interaction	GO BP	0.004	*ATG7*, *SMAD3*, *VAPB*, *ZC3H12A*, *ZNF502*
Modification of morphology or physiology of other organism	GO BP	0.008	*ATG7*, *SMAD3*, *VAPB*, *ZC3H12A*, *ZNF502*
Interaction with host	GO BP	0.010	*ATG7*,* SMAD3*, *VAPB*, *ZC3H12A*, *ZNF502*
dsRNA fragmentation	GO BP	0.014	*SNIP1*, *TARBP2*, *ZC3H12A*
Production of small RNA involved in gene silencing by RNA	GO BP	0.014	*SNIP1*, *TARBP2*, *ZC3H12A*
Production of miRNAs involved in gene silencing by miRNA	GO BP	0.015	*SNIP1*, *TARBP2*, *ZC3H12A*
Gene silencing by miRNA	GO BP	0.020	*SNIP1*, *TARBP2*, *ZC3H12A*
Positive regulation of fat cell differentiation	GO BP	0.020	*PRDM16*,* SH3PXD2B*,* ZC3H12A*
Cellular response to extracellular stimulus	GO BP	0.021	*AQP3*, *ATF4*, *ATG7*,* KLF10*,* ZC3H12A*
Cellular response to dsRNA	GO BP	0.021	*SNIP1*, *TARBP2*, *ZC3H12A*
Vasopressin-regulated water reabsorption	KEGG	0.021	*AQP3*, *LOC784058*, *RAB11A*
Posttranscriptional gene silencing	GO BP	0.022	*SNIP1*, *TARBP2*, *ZC3H12A*
Regulation of viral genome replication	GO BP	0.022	*TARBP2*, *VAPB*, *ZC3H12A*
Posttranscriptional gene silencing by RNA	GO BP	0.022	*SNIP1*, *TARBP2*, *ZC3H12A*
Positive regulation of viral life cycle	GO BP	0.022	*TARBP2*, *VAPB*, *ZNF502*
Prolactin signaling pathway	KEGG	0.022	*LOC100336962*, *PRP-VII*, *PRP14*, *PRP9*
Regulation of viral life cycle	GO BP	0.022	*TARBP2*, *VAPB*, *ZC3H12A*, *ZNF502*
Cellular response to starvation	GO BP	0.023	*ATF4,* *ATG7*, *KLF10*, *ZC3H12A*
Regulation of mitochondrion organization	GO BP	0.024	*MIEF1*,* PEMT*, *SNIP1*, *TRIAP1*, *WDR75*
Positive regulation of mitochondrion organization	GO BP	0.024	*MIEF1*, *PEMT*, *SNIP1*, *WDR75*
Cellular response to nutrient levels	GO BP	0.024	*AQP*3, *ATF4*, *ATG7*, *KLF10*, *ZC3H12A*
Regulation of fat cell differentiation	GO BP	0.024	*PRDM16*,* SH3PXD2B*, *SMAD3*, *ZC3H12A*
Response to dsRNA	GO BP	0.024	*IFNE*, *SNIP1*, *TARBP2*, *ZC3H12A*
Regulation of viral process	GO BP	0.025	*TARBP2*, *VAPB*, *ZC3H12A*, *ZNF502*
Viral genome replication	GO BP	0.025	*TARBP2*,* VAPB*, *ZC3H12A*
Regulation of protein targeting to mitochondrion	GO BP	0.025	*PEMT*, *SNIP1*, *WDR75*
Gene silencing by RNA	GO BP	0.025	*SNIP1*, *TARBP2*, *ZC3H12A*
Osteoclast differentiation	GO BP	0.025	*EPHA2*, *KLF10*, *OSTM1*
Regulation of establishment of protein localization to mitochondrion	GO BP	0.025	*PEMT*, *SNIP1*, *WDR75*
Oxidative phosphorylation	GO BP	0.025	*COX6A1*, *NDUFA12*, *TEFM*
Response to starvation	GO BP	0.025	*ATF4*, *ATG7*,* KLF10*, *ZC3H12A*
Negative regulation of defense response	GO BP	0.026	*KRT1*, *SMAD3*, *TARBP2*, *ZC3H12A*
Positive regulation of viral process	GO BP	0.026	*TARBP2*, *VAPB*, *ZNF502*
Positive regulation of establishment of protein localization to mitochondrion	GO BP	0.026	*PEMT*, *SNIP1*, *WDR75*
Positive regulation of protein targeting to mitochondrion	GO BP	0.026	*PEMT*, *SNIP1*, *WDR75*
Negative regulation of inflammatory response	GO BP	0.036	*KRT1*, *SMAD3*, *ZC3H12A*
Ribosome biogenesis in eukaryotes	KEGG	0.039	*GNL2*, *NOL6*, *WDR75*
Regulation of protein targeting	GO BP	0.041	*PEMT*, *SNIP1*, *WDR75*

We find enrichment for pathways involved in virus-host interaction, response to starvation, prolactin signalling, and other biological processes. P-value are corrected for multiple testing using Benjamini–Hochberg methodology. Enrichments represent gene ontology biological process (GO BP) or Kyoto Encyclopedia of Genes and Genomes pathways (KEGG)

## Discussion

The expression of a phenotype is not always consistent across lifespan [38]. We found that the relationship between age and hair shedding is non-linear with young cows, especially 2-year-old and 3-year-old cows, that displayed higher hair shedding scores than their older herd mates. This is in line with expectations, as young cows require increased energy expenditure associated with continued growth [39] and the new stress of lactation [40]. To a lesser extent, cows 10 to 13 years old tended to have higher hair shedding scores than young animals. A similar U-shaped relationship between age and molt date was reported in other ungulate species [8] and was reflected in the estimates of effect size from the BIF age class model (Fig. 2b). Cows are typically culled from the herd or die after 10 to 11 years of age [41, 42]. Thus, estimates of effects for cows older than 12 years reflect a selected sample. However, the early shedding estimates for these very old cows support early hair shedding as an important characteristic of longevity, especially in heat-stressed environments.

Although our results suggest a high correlation between hair shedding score breeding value across toxic fescue grazing status, we found a slightly higher heritability estimate and much larger effect of permanent environment among cattle grazing toxic fescue than those not grazing toxic fescue. Stress can sometimes increase phenotypic variation [38], which could result in the higher heritability observed among cattle grazing toxic fescue. Because repeatability is the upper bound of broad sense heritability, the disparity found in permanent environment estimates might be explained by a larger contribution of non-additive genetic effects (i.e., epistatic and dominance effects) to variation in hair shedding while grazing toxic fescue versus while not grazing toxic fescue. It is also possible that certain permanent environmental effects (i.e., physiological differences between the ability of animals to shed their winter hair) are manifest when cattle graze infected tall fescue. Most likely, the increased estimate of the permanent environment effect reflects the accumulation of physiological damage from long-term fescue toxicosis. The medial layer of blood vessels tends to be thickened in animals that suffer from fescue toxicosis, which Strickland et al. [43] linked to hyperplasia of the smooth muscle. Repeated exposure to ergovaline also increases venous contractile response, suggesting bioaccumulation [44].

Typically, measurements of the same trait across different environments that result in genetic correlations *r*_*g*_ lower than 0.80 are considered “very different” [45]. Hair shedding scores recorded while grazing toxic fescue versus while not grazing toxic fescue have an *r*_*g*_ of 0.93, which suggests minimal re-ranking of breeding values. However, the magnitude of the difference in permanent environment effects found here may justify treating hair shedding grazing and not grazing toxic fescue as separate traits in research studies that examine physiological or non-additive genetic effects. For the implementation of the American Angus NCE, we have chosen to minimize the effect of toxic grazing status by including it in the definition of contemporary groups. Many biotic and abiotic factors affect the prevalence of toxicity-inducing ergot alkaloids within forage, including moisture, reproductive status, soil nitrogen, and most notably, temperature [46]. Previous work suggests that animals must ingest a threshold level of ergot alkaloids before fescue toxicosis symptoms become evident [47]. However, in these analyses, toxic fescue grazing was treated as a binary producer-reported status in the absence of quantitative measures of ergot alkaloid levels, which may affect the interpretation of our results. Furthermore, we did not account for the effect of grazing toxic fescue in previous years.

Our enrichment results identified pathways associated with prolactin signalling, which is a well-known modulator of seasonal hair shedding and hair growth as well as milk production [4]. In 2014, Littlejohn et al. identified mutations in prolactin (*PRL*) and its receptor (*PRLR*) that cause abnormal pelage, milk production, and thermoregulation phenotypes in cattle [48]. Furthermore, low serum prolactin level is often used as an indicator of fescue toxicosis [49]. Gray et al. [22] suggested that the negative relationship that they found between calf weaning weight and dam hair shedding was due in part to differences in serum prolactin level. Our results support this conclusion. While the genetic correlation found here using all possible data between a dam’s hair shedding score and the weaning weight of her calf is moderate, it is nearly three times less than the previous estimate reported by Gray et al. [22] (*r*_*g*_ = − 0.58), which was identical to the correlation reported by Turner and Schleger [21] for a calf’s own hair shedding score and its post-weaning gain. This is likely due, in part, to our use of a slightly different phenotype. Turner and Schleger [21] used an expanded 7-point scoring system, whereas Gray et al. [22] used the same scoring system but categorized dams based on the month of the year that they first achieved a hair shedding score of 3 (about 50% shed; Fig. 1c). We also considered the relationship between hair shedding score and the maternal effect of weaning weight rather than the direct effect of weaning weight. Another possibility could be confounding environmental effects. The relationship between dam hair shedding score and calf weaning weight was also different across toxic fescue grazing statuses, and when toxic fescue grazing statuses are modelled separately the *r*_*g*_ between hair shedding score and the maternal effect of weaning weight increases relative to the *r*_*g*_ estimated using all data. This is similar to the results reported in Hoff et al. [50], where the accuracy of bovine respiratory disease (BRD) genomic prediction was higher when analysis of the data was done with data stratified by state than taken all together. The authors postulated that the discrepancy in prediction accuracy was likely due to the prevalence of different BRD-causing pathogens between environments [51]. Similarly, our results suggest that the relationship between hair shedding and other production traits may be environment- or context-specific.

In the four phenotypic regressions of calf weaning weight on dam hair shedding score, dam hair shedding while grazing toxic fescue was estimated to have the largest effect on adjusted weaning weight, but not on unadjusted weight. When contemporary grouping is fitted as a fixed effect in BLUP, the resulting contemporary group solution can be interpreted as a metric of environmental stress [52, 53]. Larger contemporary group solutions indicate a greater advantage to the phenotype from the environment, including plane of nutrition and management practices. The disparity that we found between adjusted and unadjusted weaning weight results can be explained by smaller contemporary group solutions among calves whose dams grazed toxic fescue. Indeed, the mean contemporary group solution among calves whose dams did not graze toxic fescue was 20.75 kg higher than that of those whose dams did graze toxic fescue (258.95 and 238.20 kg, respectively).

The negative genetic correlation that is often found between the maternal and direct genetic effects of weaning weight has puzzled researchers since the first large-scale national cattle evaluations, with some suggesting that it is an artefact and others that it reflects real biological phenomena [54]. We found that the magnitude of this genetic correlation varied across toxic fescue grazing statuses, with dams grazing toxic fescue showing a more negative correlation (0.63) than dams not grazing toxic fescue (0.29) (Table 2). There are several potential explanations for this result. First, the variation that we found in genetic correlations between maternal and direct weaning weight could result from sire-by-herd and sire-by-year interactions [55]. These interactions can arise via multiple avenues, including genotype-by-environment interactions, selective data reporting, and preferential management of the progeny of certain sires. If this interaction were larger in certain herds, our estimates would be skewed. Alternatively, it is possible that our results reflect the effect of fescue toxicosis on dam nutrient partitioning. Our enrichment analysis identified multiple pathways involved in response to nutrient levels, response to starvation, and fat cell differentiation, which could support this conclusion. During the initiation of lactation, mammals draw upon their own energy reserves in order to meet increased metabolic demand [56, 57], which implies genetic antagonism between maternal and direct weaning weight [37, 54]. The nutrient partitioning process is influenced by stress. For example, Rhoads et al. [58] demonstrated that decreased feed intake explains only part of the reduction in milk yield found in heat-stressed dairy cows, indicating further changes in metabolism and partitioning of nutrients in response to hyperthermia.

Although associations with a FDR less than 0.05 were detected on 20 of the 29 bovine autosomes and associations with an FDR less than 0.01 were found on seven chromosomes, one-third of the associated variants were on chromosome 5. Among these, ten variants were located near or within members of the keratin gene family. In particular, *KRT1*, *KRT3*, *KRT4*, *KRT77*, *KRT78*, and *KRT79* form a protein–protein interaction network, the orthologs of which are co-expressed in other species during the formation of intermediate filament proteins. However, it is possible that significant variants near and within keratin genes are simply an artefact of extensive linkage disequilibrium (Fig. 6b). Using the current sample, this result is difficult to disentangle.

The two most significant associations were both detected in *CEP290*. In humans, mutations in *CEP290* cause abnormal photoreceptors [59, 60]. Photoreceptors affect an animal’s ability to detect changes in seasons [61], and changes in photoreceptors could have large impacts on this function. Mutations in *CEP290* affect cilia formation, and are believed to interact with Bardet–Biedl syndrome (BBS) proteins [62]. Recently, *BBS1* was associated with local adaptation in Red Angus cattle [19]. Furthermore, the strength of these associations on chromosome 5 from 12 to 28 Mb could be due to multiple causal mutations [63] affecting multiple genes.

Many strategies have been proposed to phenotype heat stress in cattle. These methods often require the use of specialized equipment and training (e.g., body temperature, respiration rate, heart rate, and sweating rate; see [64]), or at the very least increased labor cost. Therefore, routine collection of such “gold standard” phenotypes is currently limited to use in dairy cattle or in research settings. Early summer hair shedding scoring is minimally labor intensive, since cattle need not be physically handled or processed in order to be scored. Furthermore, accurate hair shedding scoring requires a relatively small time commitment and little to no training, making it an ideal candidate for genetic evaluation at a national scale. Automated sensing technologies present an opportunity to deeply phenotype animals at large scale [65], but are not currently accessible or easily implemented by the majority of beef producers. In the future, such “gold standard” measures of heat stress could be combined with routinely collected hair shedding scores to provide a comprehensive prediction of tolerance to heat stress, fescue toxicosis, or both.

Cattle produced in sub-tropical environments account for nearly 80% of the global beef herd [66]. However, exports to South America and Australia accounted for ~ 84% of 5,333,490 total units of beef semen exported from the United States in 2019, suggesting that selection decisions made in the U.S. beef herd still have an influence on the sustainability of beef production in the global south. Furthermore, Angus genetics accounted for 86% of total semen exports in 2019 (National Association of Animal Breeders, personal communication). At an international scale, a genetic evaluation for heat stress in American Angus cattle could enable global producers to exploit elite American genetics and make faster genetic progress in production and meat quality traits while minimizing loss of environmental adaptability. Furthermore, because of the ease of phenotype collection, hair shedding scores can be collected in any temperate or subtropical environment and used in phenotypic or genetic selection for heat tolerance.

## Conclusions

We developed a prototype genetic evaluation for early-summer hair shedding in American Angus cattle in order to enable genetic selection for heat tolerance. In agreement with previous research [21, 22], we found that early summer hair shedding is moderately heritable. We also identified variants associated with biological pathways such as prolactin signalling, response to starvation, and keratin formation that contribute to genetic variation for hair shedding score. Weaning weight and hair shedding score appear to be negatively correlated. However, we found evidence for a greater impact of hair shedding score on performance for cows experiencing heat stress alone compared to cows grazing toxic fescue. Therefore, further investigation of the relationship between hair shedding and other symptoms of fescue toxicosis (such as reduced fertility) are warranted in order to determine the appropriateness of using hair shedding scores as an indicator trait for tolerance to fescue toxicosis. Exploration of the functional biology of hair shedding both on and off toxic fescue is also necessary. Finally, our results support the use of hair shedding scoring as a barometer of cow wellbeing in addition to other routinely collected phenotypes such as body condition score.

## Data Availability

Datasets supporting the conclusions of this article are available for non-commercial use via a data use agreement (DUA) with the American Angus Association.
