# Correction to: Development of a genetic evaluation for hair shedding in American Angus cattle to improve thermotolerance

**DOI:** 10.1186/s12711-021-00623-4

**Published:** 2021-03-22

**Authors:** Harly J. Durbin, Duc Lu, Helen Yampara-Iquise, Stephen P. Miller, Jared E. Decker

**Affiliations:** 1 University of Missouri, Columbia, MO 65211 USA; 2 Angus Genetics Inc., St. Joseph, MO 64506 USA

## Correction: Genet Sel Evol (2020) 52: 63 10.1186/s12711-020-00584-0

In Durbin et al. [1], the correlation between partial and full breeding values, $\rho_{p,w}^{v}$, was reported as a measure of prediction accuracy, which is not correct; the metric $\rho_{p,w}^{v}$ is the ratio between prediction accuracy in the partial dataset and prediction accuracy in the whole dataset. We have now calculated accuracy as follows:

$$ \widehat{acc}_{LR} = \sqrt {\frac{{cov\left( {\hat{u}_{w} ,\hat{u}_{p} } \right)}}{{\left( {1 - \bar{F}} \right)\hat{\sigma }_{u}^{2} }}} , $$

where $cov$ is the covariance and $\bar{F}$ is the average inbreeding coefficient of the animals in the partial dataset. The equation for $\widehat{acc}_{LR}$ is adapted from [2].

Across the 10 cross-validation iterations, the average accuracy was $\widehat{acc}_{LR}$ = 0.547 (minimum = 0.539, maximum = 0.554).
